# Mapping of fire blight resistance in *Malus* ×*robusta* 5 flowers following artificial inoculation

**DOI:** 10.1186/s12870-019-2154-7

**Published:** 2019-12-02

**Authors:** Andreas Peil, Christine Hübert, Annette Wensing, Mary Horner, Ofere Francis Emeriewen, Klaus Richter, Thomas Wöhner, David Chagné, Carolina Orellana-Torrejon, Munazza Saeed, Michela Troggio, Erika Stefani, Susan E. Gardiner, Magda-Viola Hanke, Henryk Flachowsky, Vincent G.M. Bus

**Affiliations:** 1 Julius Kühn-Institut (JKI), Federal Research Centre for Cultivated Plants, Institute for Breeding Research on Fruit Crops, Pillnitzer Platz 3a, 01326 Dresden, Germany; 2 Julius Kühn-Institut (JKI), Federal Research Centre for Cultivated Plants, Institute for Plant Protection in Fruit Crops and Viticulture, Schwabenheimer str. 101, 69221 Dossenheim, Germany; 3 The New Zealand Institute for Plant and Food Research Limited (PFR), Hawke’s Bay Research Centre, Private Bag 1401, Havelock North, 4157 New Zealand; 4 Julius Kühn-Institut (JKI), Federal Research Centre for Cultivated Plants, Institute for Resistance Research and Stress Tolerance, Erwin-Baur-Str. 27, 06484 Quedlinburg, Germany; 5 PFR, Palmerston North Research Centre, Private Bag 1600, Palmerston North, 4442 New Zealand; 6 Research and Innovation Centre, Edmund Mach Foundation, 38010 San Michele all’Adige, Italy

**Keywords:** *Malus ×robusta* 5, *Erwinia amylovora*, Fire blight resistance, QTL mapping, Orchard inoculation

## Abstract

**Background:**

Although the most common path of infection for fire blight, a severe bacterial disease on apple, is via host plant flowers, quantitative trait loci (QTLs) for fire blight resistance to date have exclusively been mapped following shoot inoculation. It is not known whether the same mechanism underlies flower and shoot resistance.

**Results:**

We report the detection of a fire blight resistance QTL following independent artificial inoculation of flowers and shoots on two F1 segregating populations derived from crossing resistant *Malus* ×*robusta* 5 (Mr5) with susceptible ‘Idared’ and ‘Royal Gala’ in experimental orchards in Germany and New Zealand, respectively. QTL mapping of phenotypic datasets from artificial flower inoculation of the ‘Idared’ × Mr5 population with *Erwinia amylovora* over several years, and of the ‘Royal Gala’ × Mr5 population in a single year, revealed a single major QTL controlling floral fire blight resistance on linkage group 3 (LG3) of Mr5. This QTL corresponds to the QTL on LG3 reported previously for the ‘Idared’ × Mr5 and an ‘M9’ × Mr5 population following shoot inoculation in the glasshouse. Interval mapping of phenotypic data from shoot inoculations of subsets from both flower resistance populations re-confirmed that the resistance QTL is in the same position on LG3 of Mr5 as that for flower inoculation. These results provide strong evidence that fire blight resistance in Mr5 is controlled by a major QTL on LG3, independently of the mode of infection, rootstock and environment.

**Conclusions:**

This study demonstrates for the first time that resistance to fire blight caused by *Erwinia amylovora* is independent of the mode of inoculation at least in *Malus* ×*robusta* 5.

## Background

The domesticated apple (*Malus domestica* Borkh.), a pome fruit species, is one of the most important fruit crops grown in temperate climate zones [1]. The most damaging bacterial disease affecting apple is fire blight, caused by *Erwinia amylovora* (Burrill) Winslow et al. [2]. Fire blight can infect its host plants via flowers (blossom blight), twigs (shoot blight) and suckers. Blossom blight leads to a reduction in crop yield, and shoot blight destroys the annual wood that bears the fruit spurs for the following season, while subsequent progression of the bacteria into large limbs or the trunk can kill the tree [3]. As most commercial rootstocks are highly sensitive to the disease, an infection of the rootstock via suckers or other pathways usually ends with death of both rootstock and scion [4]. Infestation of orchards with fire blight can cause enormous economic losses because of the necessity for elaborative sanitation measures, decreased yield, and even the eradication of trees. Van der Zwet et al. [5] reviewed economic losses in pome fruit growing over several decades. One of the last major events they listed was the fire blight outbreak in 2007 in Switzerland, associated with economic losses of around US$27.5 million. Some antibiotics, such a streptomycin, kasugamycin or oxytetracyclin, have been shown to treat fire blight effectively; however, they are registered for use in plant protection in only a few countries [6]. Their use can also reduce export opportunities when importing countries implement non-tariff trade barriers. Gianessi et al. [7] reported that US apple growers spend about US$2.8 million yearly on antibiotic sprays to prevent *E. amylovora* infection. Alternative products based on antagonists, resistance inducers or disinfecting chemicals are frequently less effective, or have a greater variation in effectiveness [8].

Another approach to control fire blight is the planting of apple cultivars that are resistant to the disease. Currently, the worldwide production of apples is dominated by a few cultivars [9], which are all more or less susceptible to fire blight. The breeding of cultivars that are both competitive in the market and resistant to fire blight is a challenge, since strong resistances are mainly found in wild *Malus* species [3, 10, 11], and their use in breeding requires several generations of pseudo-backcrosses. An understanding of the genetics of fire blight resistance is a prerequisite for target-oriented resistance breeding; to date several quantitative trait loci (QTLs) conferring various degrees of resistance to fire blight in *Malus* have been reported. The first fire blight resistance QTL, explaining up to 40% of the phenotypic variance, was mapped to linkage group (LG) 7 of ‘Fiesta’ [12, 13]. Since then, other major QTLs have been detected on LG3 of Mr5 [14, 15], on LG12 of the ornamental crab apple cultivar ‘Evereste’ and species *M. floribunda* [16] and *M*. ×*arnoldiana* [17]. A very strong QTL maps to LG10 of *M. fusca* explaining up to 66% of the phenotypic variance [18]. A candidate gene underlying the fire blight resistance QTL of Mr5 (*FB_MR5*) was identified [19] and Broggini et al. [20] confirmed the function of the *FB_MR5* CC-NBS-LRR resistance gene in transgenic ‘Gala’. Recently, Emeriewen et al. [21] reported the identification of a candidate resistance gene in *M. fusca*, which possesses a different resistance mechanism.

Furthermore, susceptibility of a given apple variety also depends on the strain of *E. amylovora* [22–24], as some have been identified that can overcome the fire blight resistance of a wild species, such as Mr5 [25, 26]. Vogt et al. [27] proposed a gene-for-gene interaction in the host-pathogen system Mr5 – *E. amylovora,* whereby a single nucleotide polymorphism (SNP) in the *avrRPT2*_*EA*_ effector leading to a change from cysteine to serine determines the difference between a compatible and an incompatible interaction. This same change in isolate Ea3049, overcoming the major QTL on LG3, enabled the detection of minor QTLs on other linkage groups [26, 28]. Recently, another gene-for-gene relationship was proposed by Wöhner et al. [29], who reported that an *E. amylovora Eop1* deletion mutant was able to cause considerable necrosis on ‘Evereste’ and *M. floribunda* 821.

Although trees are infected by *E*. *amylovora* primarily through the flowers under natural conditions in the field [30, 31], resistance phenotyping in the genetic mapping studies listed above were performed by artificial inoculation of grafted scions in the glasshouse, using either the cut-leaf [32, 33] or the hypodermic needle technique [34]. In studies comparing the resistance of shoots and flowers, Thibault and Le Lezec [35] reported a weak correlation between them, while Maroofi and Mostafavi [36] found a positive association. Nevertheless, large discrepancies between flower and shoot resistance have been found in a few cultivars. For example, ‘Reinette Clochard’ shoots were highly susceptible, but flowers had low susceptibility, while the opposite was found for ‘Blushing Golden’, ‘Mutsu’ and ‘Royal Gala’ [37].

Flowering time and climatic conditions at blooming [38, 39], length of blooming and flower age [40, 41], flower morphology [42], nectar production and composition [43, 44] and volatile organic compounds emission [31] may influence flower blight incidence. To date, it is unclear whether resistances to fire blight of shoots and flowers are governed by the same mechanism, or if the observed differences are based mainly on differing environmental conditions. Here, we report the mapping of fire blight resistance in Mr5 following separate artificial inoculations of flowers and shoots in the field and the glasshouse, respectively, in two countries. The F1 generation from a cross of ‘Royal Gala’ × Mr5 was planted in New Zealand using a single rootstock, while the progeny from a cross of ‘Idared’ × Mr5 was planted in Germany on two different rootstocks. Part of the same ‘Idared’ × Mr5 population had previously been used for genetic mapping of resistance to fire blight by evaluation of shoot infections under glasshouse conditions [14, 26, 28, 45].

## Results

### Flower phenotyping

**Fig. 1:**
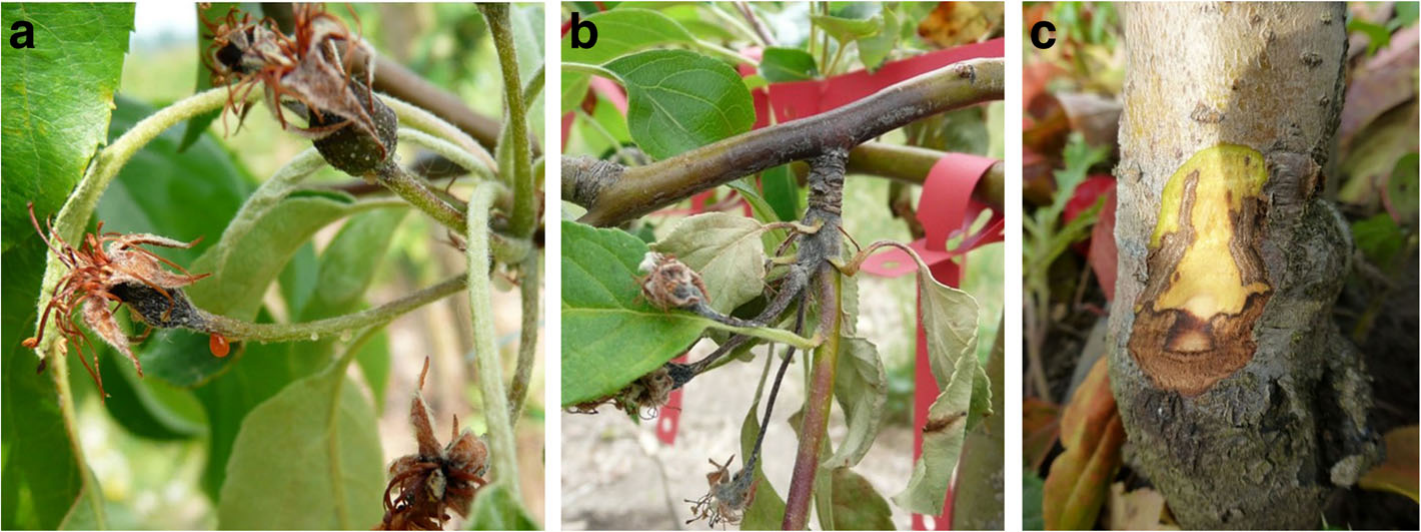
Fire blight symptoms of apple flowers, shoot and rootstock. **a** Symptomatic flower stem/fruit stem (scoring value 2) with ooze **b**) Shoot necrotic lesion < 5 cm (scoring value 5) **c**) Rootstock blight, showing the sharp separation between healthy and symptomatic tissue

The harmonized classification for flower infection by fire blight (Table 1; Fig. 1a, b) facilitated the assessment of symptoms in both Germany and New Zealand.

**Table 1:** Ranking systems used in New Zealand and Germany for phenotypic assessment of apple floral clusters after inoculation with *Erwinia amylovora*

New Zealand				Germany
Symptom description	Class	Harmonized	Class	Symptom description
healthy	0	0	0	healthy
			1	possible floral infection
floral infection	1	1	2	clear floral infection
flowers and peduncle infected	2	2	3	flowers and peduncle infected
flowers and bourse infected	3	3	4	flowers and bourse leaves infected
			5	flowers and bourse infected
floral cluster, bourse and bourse shoot infected	4	4	6	floral cluster, bourse and possibly shoot infected
necrosis spread < 5 cm	5	5	7	necrosis spread < 5 cm
necrosis spread > 5 cm	6	6	8	necrosis spread > 5 cm

In Germany, a total of 58 flower clusters on Mr5 were inoculated between 2011 and 2013, but not a single cluster exhibited symptoms, whereas the inoculation of 297 floral clusters in the 2015–2017 period resulted in low infection rates, ranging from 0.08 to 0.28 (Table 2). The scores for ‘Idared’ in the same 2015–2017 period ranged from 2.01 to 3.71 (Table 2).

**Table 2:** Total number of trees and number of flowering trees and flower clusters, the annual mean fire blight infection score of the progeny and parents of an ‘Idared’ × *Malus* ×*robusta* 5 (Mr5) population inoculated with *Erwinia amylovora* strain Ea222_JKI in the period 2011–2017 in Germany. For the progeny, the percentage of progeny rated Class 0 (i.e. no infection) is presented for each year

Year^a^	Progeny						Parents					
				# flower clusters inoculated	Mean score^b^		‘Idared’			Mr5^c^		
	#	# trees flowering				% Class 0	# trees	# flower clusters inoculated	Mean score^b^	# trees	# flower clusters inoculated	Mean score^b^
		Total	Mapping population									
2011	239	188	91	1573	1.86a	34.0	0			1	3	0
2012	233	228	111	3367	0.95c	35.1	0			1	20	0
2013	173	154	65	2722	0.47e	37.0	0			1	35	0
2015	150	148	126	2546	0.75d	29.1	10	112	2.01c	5	58	0.28a
2016	150	59	46	325	1.11bc	61.0	9	69	3.71a	8	69	0.10ab
2017	150	146	125	1982	1.37b	26.0	10	100	2.81b	9	112	0.08b

^a^Progenies from trial 2011–2013 were grafted on ‘M9’ and progenies from trial 2015–2017 were grafted on ‘B9’

^b^Different letters indicate significantly different means at α = 0.05

^c^Data from 2011 to 2013 were not included in analysis

**Fig. 2:**
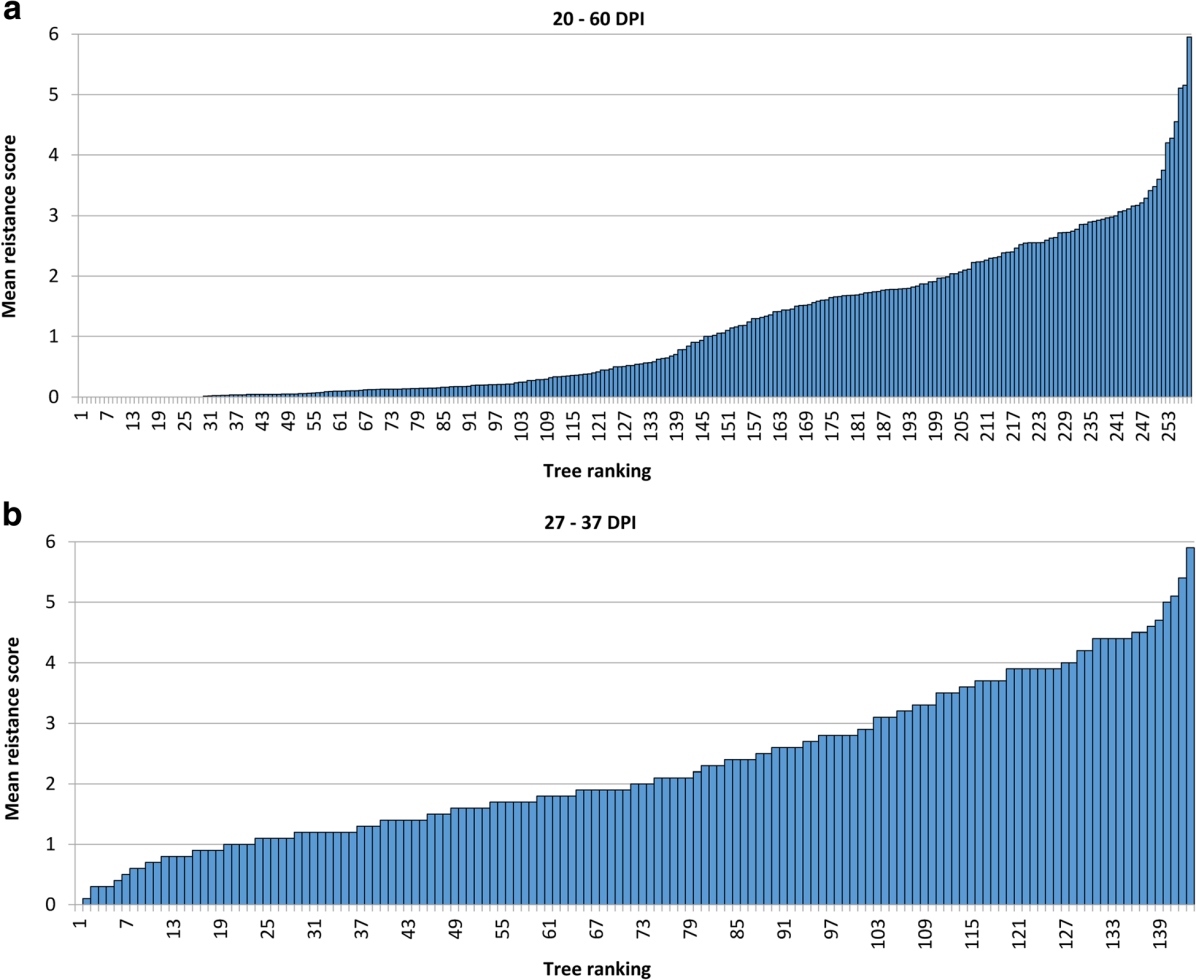
**a** Ranking of genotypes in the German ‘Idared’ × *Malus ×robusta* 5 population ordered by degree of infection 20–60 days after inoculation of floral clusters with *Erwinia amylovora* isolate Ea222_JKI. All data (2011 to 2013 and 2015 to 2017) available for a genotype were averaged for the mean score. Inoculated genotypes are listed in Additional file 4: Table S1. **b** Ranking of genotypes in the New Zealand ‘Royal Gala’ × *Malus ×robusta* 5 population ordered by degree of infection 27–37 days after inoculation of floral clusters with *E. amylovora* isolate Ea236. Ten clusters were inoculated on each genotype. DPI = days post-inoculation

In total, 12,515 flower clusters were inoculated on the seedling progeny over the 6 years of the experiment, with 228 (95.4%) out of the initial 239 ‘Idared’ × Mr5 progeny on ‘M9’ rootstock, and 148 (98.7%) progeny out of 150 on ‘B9’ rootstock inoculated at least once in the periods 2011–2013 and 2015–2017, respectively (Table 2). The number of inoculated flower clusters per genotype is shown in Additional file 4: Table S1. The highest rate of infection was recorded in 2011 when the mean disease score was 1.86, and the lowest score (mean of 0.47) was recorded in 2013 (Table 2). Each year, about one-third of the progeny did not show any symptoms, except for 2016 when the proportion was 61.0%, with 29 (11.2%) out of 258 genotypes not expressing any symptoms in any year. The highest mean score for an individual genotype was 6.0 in 2012, 3.2 in 2013, 3.0 in 2015, 4.0 in 2016 and 6.0 in 2017. For 147 (57.0%) progeny, the mean score was 1 or lower and the highest average score was 5.95 (Fig. 2a). Additional file 1: Figure S1 shows the proportion of the seven rankings for each progeny.

Since not all progeny were consistently inoculated in every year due to alternate flowering and/or loss of trees, an additional evaluation of the year effect was performed on the genotypes that were inoculated in every year of each of the periods 2011–2013 and 2015–2017, and combined for the 2011–2017 period. Significant year effects were observed (Additional file 2: Figure S2a-c), as well as significant genotype effects (Additional file 3:Figure S3a-c) in this analysis. Flower clusters of 131 genotypes of this German population were phenotyped at least once in each of the 2011–2013 (genotypes grafted on ‘M9’) and 2015–2017 (genotypes grafted on ‘B9’) periods, which showed a correlation of r = 0.76 for the mean scores of the genotypes between the two time periods.

The mean disease scores from the single-year phenotyping of the New Zealand ‘Royal Gala’ x Mr5 family of 143 plants ranged from 0 to 5.95. This population displayed a somewhat higher mean rate of infection (2.3) than the German family, with only one progeny of the NZ family not showing any symptoms (Fig. 2b), and only 23 (16%) exhibited a mean infection score of 1 or lower.

### Shoot inoculation and assessment

**Fig. 3:**
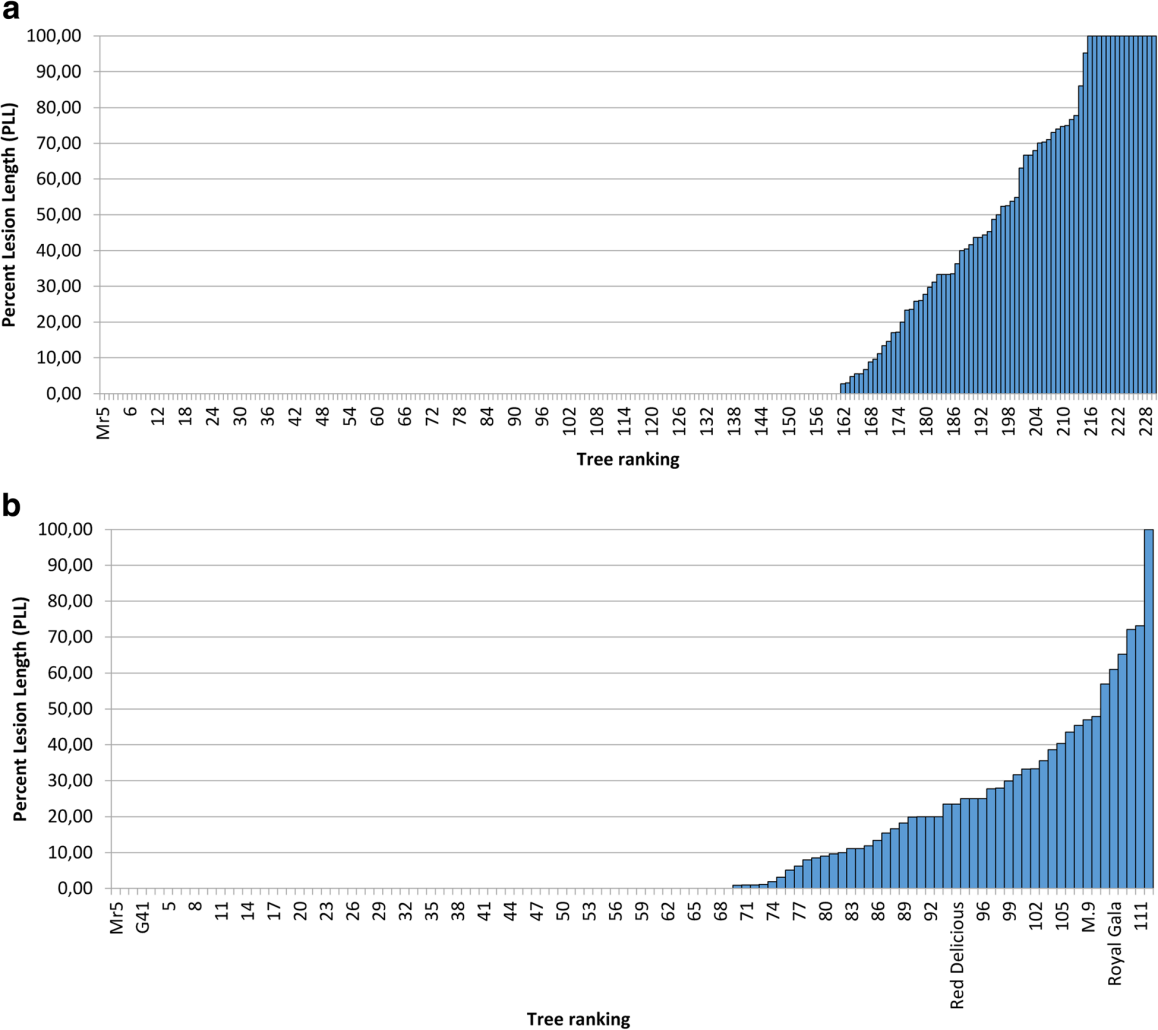
**a** Ranking of the percent lesion length (PLL) of *Malus ×robusta* 5 (Mr5) and 230 progeny from the German ‘Idared’ × Mr5 population 24 days after artificial shoot inoculation in the orchard in 2012. **b** Combined ranking of the PLL of 112 progeny from the New Zealand ‘Royal Gala’ × *Malus ×robusta* 5 population 28 days after artificial shoot inoculation in the glasshouse in 2012 and 2013

The ranking of the mean PLL for each of the 230 progeny of the ‘Idared’ × Mr5 population in 2012 displayed a strong bias towards resistance (Fig. 3a). While inoculated scions of 15 progeny showed 100% PLL, inoculated shoots of 70% of the progeny, together with Mr5, did not exhibit any necrosis, which resulted in a mean PLL for the progeny of 16.2%. The correlation between the mean scores of flowers and PLL of shoots in the orchard was r = 0.61.

The ranking of the 109 ‘Royal Gala’ × Mr5 progeny in the glasshouse in New Zealand had a very similar bias to that in the German family (Fig. 3b), with the majority of the genotypes (61%) exhibiting no symptoms and a mean PLL of 9.1%. A weak year effect was observed, although the ‘None’/‘Some’ test showed that infection was consistent over both years, but no significant correlation between flower and shoot resistance was found (Horner et al. 2014).

### Analysis of rootstock blight

Of the 83 samples from trees exhibiting symptoms typical of rootstock blight (Fig. 1c) in autumn 2012 and screened by PCR for the presence of *E. amylovora*, 24 tested positive for rootstock infection with fire blight. The average score of these 24 progenies after inoculation of flowers in 2012 was 1.16, with a maximum of 4.12. The average PLL after shoot inoculation of the same genotypes was 23.8, with a maximum of 100. The correlation of scores and PLLs was r = 0.70. Four out of the 24 genotypes that tested positive for rootstock infection had previously exhibited no symptoms either in the scion or in any floral cluster following inoculation with Ea222_JKI.

### Genetic mapping of flower resistance to fire blight

**Fig. 4:**
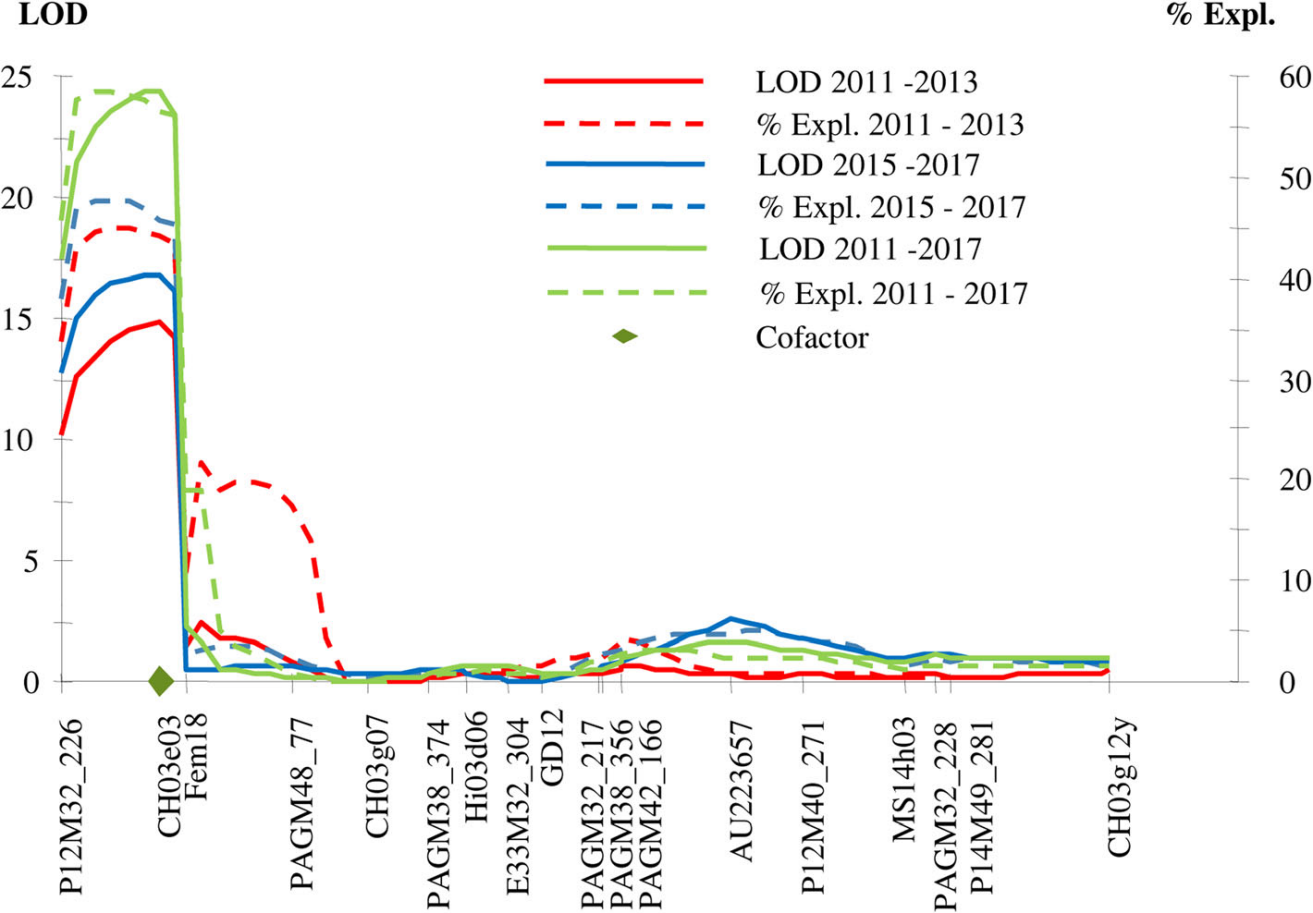
Logarithm of odds (LOD) score and the percentage of the phenotypic variation explained (% Expl.) by the genetic markers after MQM mapping of disease infection data following artificial flower inoculation with Ea222_JKI along linkage group 3 of *Malus* ×*robusta* 5 (Mr5) in the German ‘Idared’ × Mr5 population based on the average median of the genotypes for the periods 2011–2013, 2015–2017 and 2011–2017. Cofactor = marker with the highest LOD score after interval mapping was set as co-factor

A subset of the ‘Idared’ × Mr5 family (Table 2) was genotyped for the genetic mapping of the flower resistance in the six-year experiment in Germany. The scoring data for the individual years, as well as the average scores over both the 2011–2013 and 2015–2017 periods, and all years were used for Kruskal-Wallis analysis, permutation tests, and interval and MQM mapping. To test the robustness of the homogenized ranking scale, interval mapping was performed with both the original (scale 0–8) and the harmonized ranking (scale 0–6) for each year separately, the 2011–2013 and 2015–2017 periods, and both periods together. All the separate analyses displayed the same single major QTL identified on LG3 for both 3-year periods as well as for all 6 years, with the LOD score ranging from 14 to 23 (Fig. 4). No other significant QTLs for flower resistance to fire blight were detected.

**Fig. 5:**
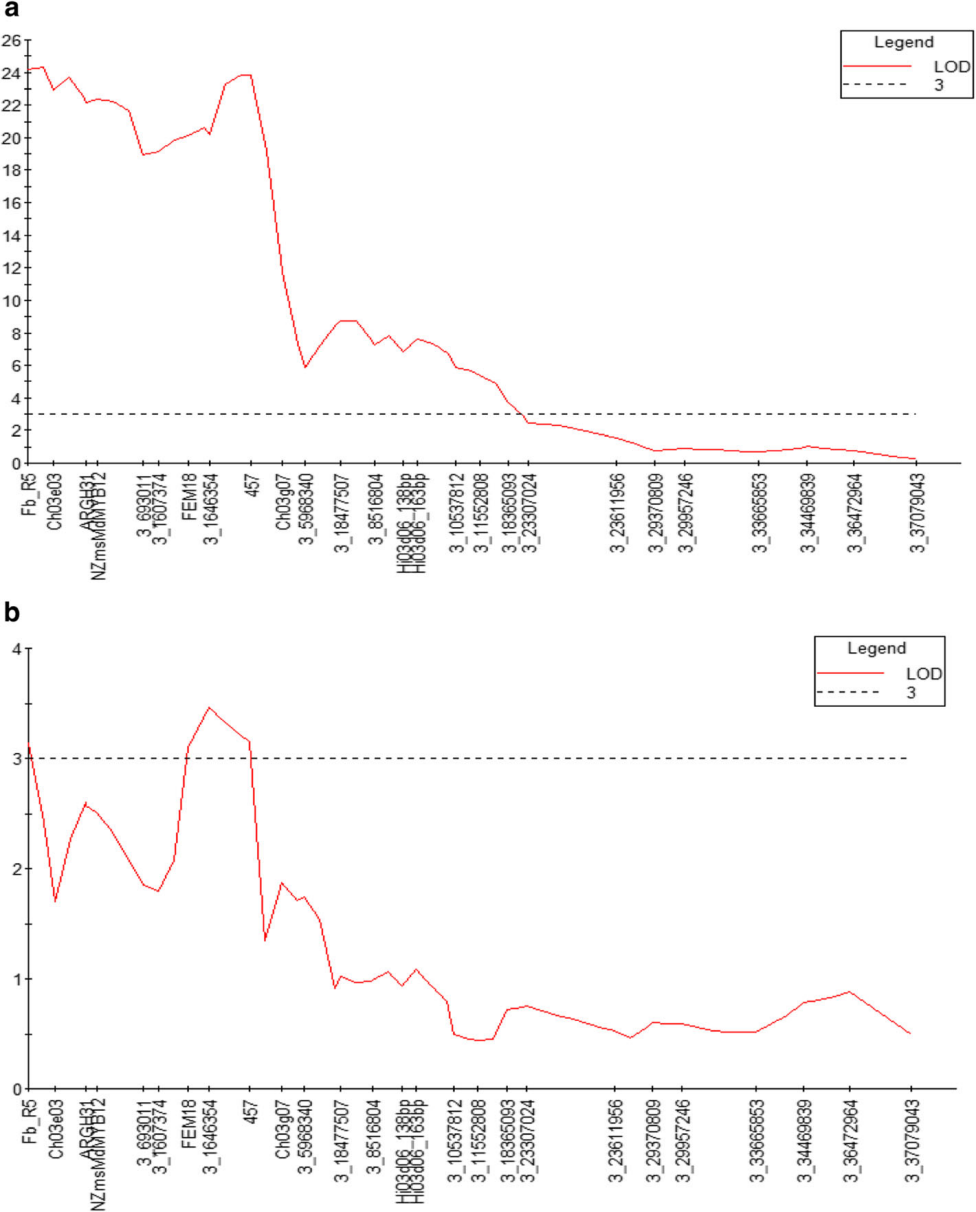
Interval mapping of quantitative trait loci (QTLs) on linkage group 3 of Malus ×robusta 5 for control of fire blight resistance following inoculation of flowers in the field (**a**) and shoots in the glasshouse (**b**) of the New Zealand ‘Royal Gala’ × *Malus* ×*robusta* 5 population with Erwinia amylovora isolate Ea236. LOD = logarithm of odds

In the New Zealand ‘Royal Gala’ × Mr5 population subjected to floral inoculation with Ea236 in the field, IM revealed a QTL controlling resistance to fire blight in the same position as that identified in the ‘Idared’ × Mr5 population inoculated with Ea222_JKI in Germany, albeit in a somewhat larger interval (Fig. 5). The peak of the LOD curve positioned over markers Fb_R5 and CH03e03 was > 24. No other significant QTLs for control of fire blight resistance were observed in this population, using either IM or Kruskal-Wallis analysis.

### Genetic mapping of shoot resistance to fire blight

Kruskal-Wallis analysis of PLL data following shoot inoculation of the ‘Idared’ × Mr5 population demonstrated marker-trait association for LG3, with the highest K value obtained for marker CH03e03. Genotypes with the 185 bp allele of CH03e03 in coupling phase with resistance exhibited a mean PLL of 0.2% compared with a mean PLL of 37.5% exhibited by genotypes expressing the 207 bp allele in repulsion phase to resistance. Interval mapping resulted in a LOD of 9.8 for CH03e03, well exceeding the threshold of 2.8 (data not shown).

As genotype data were not available for all the shoot-inoculated plants of the ‘Royal Gala’ × Mr5 population in the glasshouse, IM was performed on a subset of 60 plants. This analysis revealed a broad, low-significance QTL on LG3 of Mr5, which aligned with the QTL identified by Kruskal-Wallis analysis in the ‘Idared’ × Mr5 population (Fig. 5). No other associations of phenotype with genotype were identified in the ‘Royal Gala’ × Mr5 population.

## Discussion

Fire blight resistance has long been thought to be quantitative, and a number of QTLs from a range of sources have been reported to date [46]. However, mapping of fire blight resistance in *Malus* has been performed exclusively with data generated from artificial shoot inoculations [12, 14, 16–18]. A reasonable justification for using shoot blight severity as a measurement for resistance according to Harshman et al. [47], is that shoot infection generally leads to structural damage and ultimately economic losses over time. However, the common practice of utilizing only shoot inoculation to map fire blight resistance has resulted in a limited understanding of the genetic control of floral fire blight resistance: breeders have been uncertain whether genetic markers for Mr5 resistance to fire blight derived from QTL mapping of shoot inoculation data [45] are also valid for marker-assisted selection (MAS) for resistance following floral infection, which is the usual point of entry in the field. Also, optimized infection conditions for the pathogen in the glasshouse may overestimate susceptibility, whereas artificial inoculation in the orchard does not guarantee infection or progress of the disease if weather conditions critical for fire blight development are suboptimal. In the latter case, we tried to improve flower infection conditions by covering the flowers to protect them from unfavorable weather conditions both in Germany and New Zealand.

Reliable phenotypic evaluation protocols critical for the genetic mapping of fire blight resistance were adopted for our study and the disease scoring was harmonized to ensure comparability across the two different populations in the two countries. While there were many confounding factors associated with the geographic distance between the two studies (differences in *E. amylovora* isolates; time of year; inoculation techniques; number of years), the phenotyping results were very similar in general, and resulted in identification of the same LG3 QTL on the Mr5 genome.

In the German experiment, a correlation of r = 0.61 was obtained between the results of floral and shoot inoculations, which was higher than the weak correlation (0.25 < r < 0.44) found by Thibault and Le Lezec [35] for ‘Gala’. This may be explained by the large genetic effect of the Mr5 QTL, whereas the results for ‘Gala’, a susceptible cultivar, might have been influenced mainly by environmental conditions. The correlation between flower resistance of progeny grafted onto both ‘M9’ and ‘B9’ rootstocks was high (r = 0.76), in spite of the climatic conditions during inoculation being different during the different periods of observations. The significant year effects on the mean scores over 6 years of floral infection on the ‘Idared’ × Mr5 progeny and the susceptible parent ‘Idared’ provide further evidence that environmental conditions influence fire blight infection. Van der Zwet et al. [48] argued that weather is one of the most important factors contributing to fire blight incidence in its host. Although these environmental effects were not investigated in detail, it is plausible that conditions were more favorable for *E*. *amylovora* in 2011, when the highest infection rate was observed, and less favorable in 2013, the year with the lowest infection rate. However, as loss of susceptible trees also occurred, the data set of second and third year inoculations has an additional bias towards more resistant trees. Typical differences in reactions among genotypes to this pathogen have also been previously reported for shoot blight evaluations in the glasshouse [18].

*E. amylovora* is capable of migrating through healthy scion tissue into the rootstock, causing rootstock blight [49], which can lead to the death of a tree grafted on susceptible rootstocks [50]. Although ‘B9’ is susceptible to fire blight, it appears to be resistant as a rootstock, leading LoGiudice et al. [51] to suggest that it suppresses the multiplication of *E. amylovora.* In our study, a good proportion of the trees of the ‘Idared’ × Mr5 population grafted on ‘M9’ displayed conspicuous rootstock blight symptoms, which was confirmed by PCR on rootstock samples, even though in some cases the scion genotype had not exhibited disease symptoms. However, the same genotypes grafted onto ‘B9’ exhibited no rootstock blight. To prevent tree death due to fire blight infection, a combination of both resistant rootstock and scion appears to be necessary and this should be further investigated.

QTL mapping using phenotype data sets for each of the 6 years of artificial flower inoculation with *E. amylovora* in the ‘Idared’ × Mr5 population, and for the single year in the ‘Royal Gala’ × Mr5 population, resulted in the detection of a single major QTL for control of floral fire blight resistance on LG3 of Mr5, although the QTL was slightly broader in the ‘Royal Gala’ × Mr5 population. This flower resistance QTL appears to be the same as the shoot blight resistance QTL from Mr5 reported previously [14, 15, 45] and was re-confirmed here through the phenotyping of subsets of these populations for this trait, both in the field in Germany and in the glasshouse in New Zealand. Taken together, results of the current study provide strong evidence that fire blight resistance in Mr5 is controlled by the same major QTL on LG3, independent of the infected host tissue and a range of environmental conditions during infection.

Since the QTL explains a large proportion of the phenotypic variation [14, 15], it can be regarded as a major-effect gene: such genes generally tend to be less influenced by the environment. While our study confirmed that the LG3 QTL for shoot resistance applies to flower resistance, the low correlation between the shoot and flower screening methodologies identified in a preliminary analysis of the NZ ‘Royal Gala’ × Mr5 population [52] suggests that other undetected resistance factors may be present.

The robustness of the LG3 QTL under different phenotyping conditions makes it a solid choice for development of high-throughput markers for MAS for fire blight resistance derived from Mr5. We developed the Taqman™ Fb_R5 marker [53] to provide a cost-effective alternative to the *MxdRLP1* high-resolution melting (HRM) marker developed earlier, following mapping of fire blight resistance after shoot inoculation in a ‘M9’ × Mr5 population [45]. The Fb_R5 marker has been employed for MAS in populations from the PFR rootstock breeding programme during the past year.

## Conclusions

We have clearly demonstrated that the strong QTL controlling fire blight resistance in the flowers of Mr5 is located in the same position on LG3 as the one identified through shoot infection. Data gained from the method of inoculating flowers with *E. amylovora* led precisely to the detection of the locus on LG3 in each year of inoculation under two different environments. Although with this method, infection of tissue through micro wounds close to inoculated flowers cannot be excluded, it is highly unlikely that the results obtained are biased in favour of shoot inoculation through these micro wounds rather than by flower inoculation, especially, as it is impossible to have wounds on the same genotypes each year and in both countries, which could have led to the detection of the exact same locus.

The position of the QTL has been demonstrated to be independent of environment through the use of two progenies derived from crosses of Mr5 with different fire blight-susceptible apple parents grown and phenotyped in Germany and New Zealand. In Germany, the QTL was stable over 6 years of floral inoculation in the field as well as independent of rootstock. Nevertheless, this study is limited by the fact that flower inoculation of the NZL population was done only once and infection in the orchard depended on favorable conditions for the pathogen, but even under best conditions, infection can still not be guaranteed. Also, the distribution of the pathogen in asymptotic tissue cannot be excluded. Asymptomatic tissue could be the source for rootstock blight through internal migration of the pathogen leading to the death of the tree or the source for pathogen delivery to other trees. Future studies should determine the correlation between resistant genotypes and asymptomatic tissue if feasible.

To our knowledge, this is the first report of the identification of a QTL controlling fire blight resistance in flowers. Validation of the exact genetic control of this floral resistance is still required to ensure the effective application of MAS. As the strongest fire blight QTLs tend to be found in wild *Malus* species, we anticipate that shoot phenotyping will remain a suitable proxy for flower resistance phenotyping in introgressing these other QTLs into domesticated apple and for QTL mapping as a precursor to MAS for durable resistance.

## Methods

### Plant material

Two independent segregating populations were derived from crosses between a susceptible apple cultivar and the fire blight resistance donor Mr5. The German population consisted of 258 progeny derived from an ‘Idared’ × Mr5 cross, which were grafted onto ‘M9’ rootstock and planted in the Kirschgartshausen research orchard of the Julius Kühn-Institut (JKI) in 2010. Additional trees of a subset of 150 genotypes of the population grafted onto Budagovski9 (‘B9’) rootstock were planted there in 2014. Grafted plants of the respective parents were included as controls in both years, of which the susceptible control ‘Idared’ planted in 2010 did not establish. A subset of 140 progeny of the same population had been used previously to establish the genetic map [28], which was used for QTL mapping. The New Zealand population consisted of 287 progeny from a ‘Royal Gala’ × Mr5 cross grafted on ‘M27’ rootstock and planted in the Hawke’s Bay research orchard of The New Zealand Institute for Plant and Food Research Limited (PFR). Evaluation for floral fire blight resistance was performed on 143 progeny of the population. A subset of 112 of these (78%) were grafted onto ‘M9’ rootstock, with up to 10 replicates/genotype, and raised in the glasshouse for shoot inoculation.

Cultivars and the wild *Malus* accession Mr5 are maintained in the NZ and JKI germplasm collections since decades; seeds of populations were obtained from crosses in the respective institute and rootstocks for grafting were obtained from nurseries.

### Inoculum

In Germany, strain Ea222_JKI (Internal collection of *E. amylovora* strains held at JKI, Quedlinburg) from frozen stock was used for *E. amylovora* inoculation. The frozen stock was prepared by pelleting cells, obtained from liquid cultures grown overnight at 28 °C, at 140 rpm in StI medium (Roth, Karlsruhe) and re-suspending the cell pellets in sterile 15% v/v glycerol for storage at − 80 °C until use. Inoculum was prepared by slowly thawing the cell suspensions and diluting them with sterile water to a final concentration of 1 × 10^9^ colony forming units (cfu)/mL using photometric measurement and validated by live cell counts from dilution plating on St1 agar. *E. amylovora* strain Ea222_JKI [27] was used previously to detect several major resistance QTLs in *Malus* [14, 17, 18], including the QTL of Mr5 on LG3 after artificial shoot inoculation in the greenhouse.

In New Zealand, strain Ea236 (International Collection of Micro-organisms from Plants (ICMP), Manaaki Whenua, Landcare Research New Zealand Ltd) was grown on King’s B agar [54] for 48 h at 26 °C from stock held at − 80 °C. Inoculum was prepared freshly by collecting bacteria from the medium and suspending them to a final concentration of 1 × 10^9^ cfu/mL in a phosphate buffered saline (PBS) solution at pH 7.2 using photometric measurement and validated by live cell counts from dilution plating on King’s B medium.

### Flower inoculation and phenotyping

In Germany, flower inoculation of the ‘Idared’ × Mr5 population in the Kirschgartshausen orchard was undertaken from 2011 to 2013 for the progeny grafted on ‘M9’ rootstock, and from 2015 to 2017 for the progeny grafted on ‘B9’ rootstock. Young flowers were inoculated with the Ea222_JKI suspension using an EcoSpray TLC-sprayer (Roth, Karlsruhe). Each flower cluster was sprayed with approximately 100 μL inoculum and bagged with a plastic bag, which was removed 5 days after inoculation. Disease assessment was performed 20–60 days after inoculation.

At the PFR Hawke’s Bay site, 10 floral clusters on a single tree of each genotype were inoculated in the orchard with isolate Ea236 in the period 2–30 September 2009 as the flower clusters reached the standard cluster maturity, using a DeVilbiss atomizer No. 15 with a final load of 10^5.6^ cfu/flower. Each floral cluster was bagged with a plastic bag, and then covered in aluminum foil to shade the flowers to prevent overheating in direct sunshine. Bags were removed 3 days after inoculation and disease assessment was performed at 9–12 and 27–37 days after inoculation.

Infection of the floral clusters was assessed in a seven-class in-house ranking system in New Zealand and in a nine-class in-house system in Germany. To harmonize the datasets, the German 0–8 scale was transformed to the New Zealand scale 0–6 (Table 1). In Germany, each season, the trees were sanitized by extensive pruning following evaluation. Symptomatic tissue was removed and affected shoots were cut back deep into the healthy regions. Figure 1a, b shows fire blight symptoms after inoculation of flowers.

### Shoot inoculation and phenotyping

In Germany in 2012, shoot inoculation was performed on 230 genotypes in the orchard to compare results obtained in glasshouse experiments with field data. Inoculum of *E. amylovora* Ea222_JKI was prepared as described above and sprayed onto a pair of scissors that were used to cut the tips of the two youngest leaves on each of three young shoots per tree. Disease severity was evaluated 24 days post inoculation and calculated as percent lesion length (PLL) of the shoot, by dividing the symptomatic shoot length by the total length of shoot and multiplying by 100.

In New Zealand, a subset of 112 of the 143 florally inoculated plants was also assessed for shoot resistance. Progeny were carefully selected to ensure that the population subset to be screened had plant numbers that fell evenly into each of the floral resistance classes of 0–6. Up to 10 replicates of actively growing shoots (minimum length of 16 cm) from each of the 112 progeny, and replicated parent and control plants (‘Royal Gala’, Mr5, ‘M26’, G41, ‘Red Delicious’ and ‘M7’) grown on ‘M9’ rootstock were inoculated in both 2012 and 2013 by cutting the tips of the two uppermost unfolded leaves with scissors that had been dipped in to a 10^9^ cfu/mL suspension of *E. amylovora*. The inoculated plants were incubated in the glasshouse, with the length of necrosis of each shoot and total shoot length being measured 28 days post-inoculation (DPI). A mean length of necrotic shoot was estimated for each plant using Genstat’s CENSOR procedure to account for the possibility that infections that had completely infected the shoot, would have grown longer on a longer shoot. Disease severity was calculated as PLL. Progeny were also classified into two groups depending on whether any of the replicates became infected (‘Some’) or not (‘None’). This was done for both the 2012 and 2013 data, creating a 2 × 2 classification.

### Rootstock blight verification by polymerase chain reaction

During field assessment in Germany, a number of trees developed symptoms of rootstock blight (Fig. 1c) despite pruning. This was especially prevalent during summer/autumn 2012. To confirm the presence of *E. amylovora*, a sample was taken from 83 trees displaying such symptoms, at the transition between healthy and symptomatic tissue from the graft union. Bacteria were washed from the tissue by shaking several small slivers in 1 mL water for 10 min. A 100-μL aliquot of the resulting suspension was used to inoculate 1 mL StI medium cultures each for analysis by polymerase chain reaction (PCR). Sample preparation and PCR were performed with the primer combination #149 (CCGAAGAACGATTGCACTAC) and #150 (CGGTTAGTTAGCGCAGTCTC) as described by Wensing et al. [55]. Sample dilutions from 10- to 1000-fold were tested for each sample, to remove traces of PCR inhibitory compounds.

### DNA extraction and genotyping of ‘Royal Gala’ × Mr5 population

Genomic DNA was extracted from young freeze-dried leaves harvested from 109 trees of the ‘Royal Gala’ × Mr5 population and its parents, using the QIAGEN DNeasy® Plant Mini Kit (QIAGEN GmbH, Hilden, Germany) according to the manufacturer’s protocols. This DNA was amplified and hybridized to the apple and pear Infinium® II 9 K SNP array [56] following the Infinium® HD Assay Ultra protocol (Illumina Inc., San Diego, USA) and scanned using an Illumina HiScan. Data were analyzed using Illumina® GenomeStudio v 1.0 software Genotyping Module, with a *GenCall* Threshold of 0.15. Additional simple sequence repeat (SSR) markers mapping at the top of LG3 were screened over the population to enable comparison of the results in the present study with those reported by Gardiner et al. [45]. Fb_R5 is a Taqman™ marker developed from the sequence around the *MxdRLP1* marker [45] and is used routinely for marker-assisted selection for fire blight resistance derived from Mr5 in PFR breeding programmes [57]. The Fb_R5 marker was developed from the FB-MR5-NZsnEH034548_R249 SNP [58] and is available as a custom Taqman™ assay (assay AH21B92; Thermo Fisher Scientific).

### Genetic mapping and statistical analyses

The Mr5 map established by [28] was used as a template for QTL mapping in the ‘Idared’ × Mr5 population and a new genetic map was generated for Mr5 in the ‘Royal Gala’ × Mr5 population using SNP array data and supplementary markers from LG3 of the ‘M9’ × Mr5 map [45].

MapQTL®5 [59] was used throughout this study for Kruskal-Wallis analyses, interval mapping (IM) and multiple QTL model (MQM) mapping. Thresholds were determined by permutation tests with a significance level of α = 0.05. The marker with the highest logarithm of odds (LOD) score close to a QTL was included as co-factor for MQM mapping.

Statistical analyses to determine whether trial year caused significant differences in the average score, at a significance level of α = 0.05, were performed using SAS GLIMMIX (Generalized Linear Mixed Model) procedure (SAS-Institute 2013, [60]. Therefore, the averages of the mean scores of all clusters for a single year were calculated.

## Supplementary information

**Additional file 1:** **Figure S1**. Ranking of genotypes in the German ‘Idared’ × *Malus ×robusta* 5 population ordered by degree of infection 20–60 days after inoculation of floral clusters with Ea222_JKI. All data (2011 to 2013 and 2015 to 2017) available for a genotype were averaged for the mean score. Different colours indicate the percentages of the scores 0–6 for the floral clusters of a genotype.

**Additional file 2:** **Figure S2**. Significant differences between mean resistance scores of individual years in the German ‘Idared’ × *Malus ×robusta* 5 population. Only genotypes tested in all years of the respective period were utilized for the analysis. The significance level is α = 0.05. **a.** Period 2011 to 2013. **b.** Period 2015 to 2017. **c.** Period 2011 to 2017

**Additional file 3:** **Figure S3.** Significant differences between mean resistance scores of genotypes in the German ‘Idared’ × *Malus ×robusta* 5 population. Only genotypes tested in all years of the respective period were utilized for the analysis. Genotypes without any symptoms in the respective period were removed from analyses, because of the lack of standard deviation. Different letters on the right side indicate significanet differences at a level of α = 0.05. **a.** Period 2011 to 2013. **b.** Period 2015 to 2017. **c.** Period 2011 to 2017

**Additional file 4:** **Table S1.** Number of inoculated flower clusters per genotype, year and scoring results.

## Data Availability

The data that support the findings of this study are available from the authors upon reasonable request.

## Abbreviations

bp: base pairs
cfu: colony forming units
F1: 1 filial generation
GLIMMIX: Generalized linear mixed model
IM: Interval mapping
JKI: Julius Kühn-Institut
LG: Linkage group
LOD: Logarithm of the odds
MAS: Marker assisted selection
mL: milli litre
MQM: Multiple QTL model
Mr5: *Malus* ×*robusta* 5
NZ: New Zealand
PBS: Phosphate buffered saline
PCR: Polymerase chain reaction
pH: potentia Hydrogenii
PLL: Percentage lesion length
QTL: Quantitative trait locus
rpm: rounds per minute
SNP: Single nucleotide polymorphism
V/V: Volume per volume
