# Supplementary figures and images for: Mapping of fire blight resistance in Malus ×robusta 5 flowers following artificial inoculation
Source: BMC Plant Biol. 2019 Dec 2;19:532. doi: 10.1186/s12870-019-2154-7 (PMC6889339; doi:10.1186/s12870-019-2154-7)

Scoring value: 0 1 2 3 4 5 6

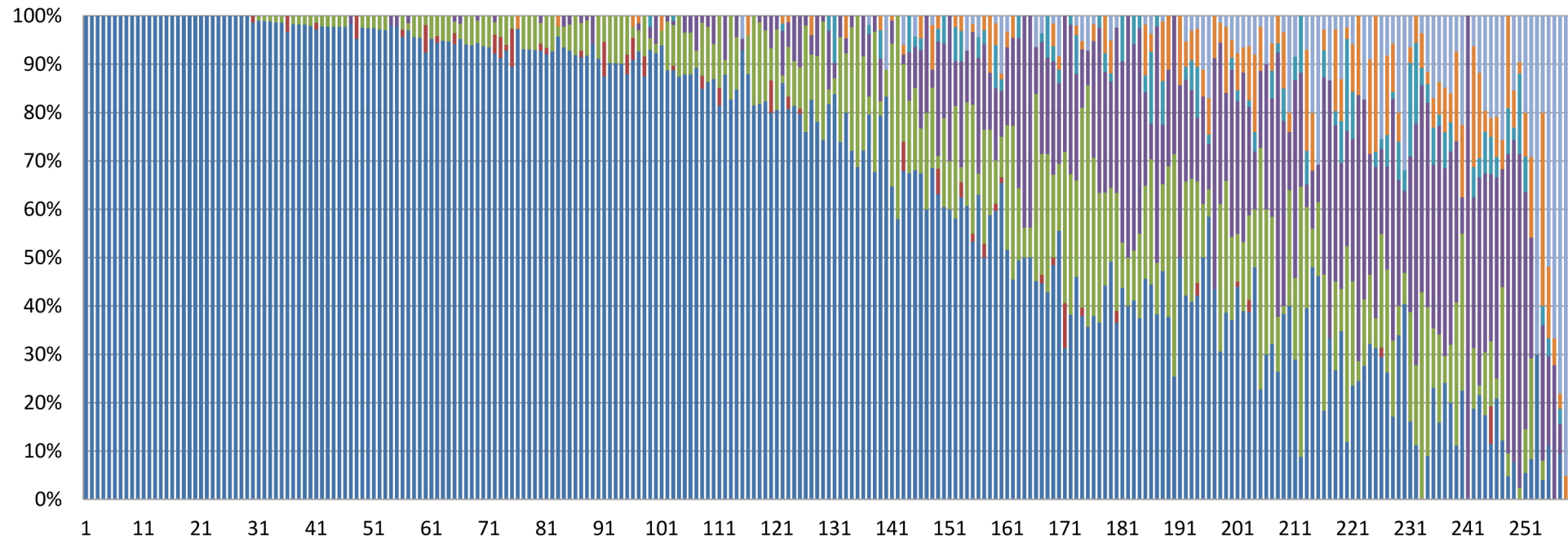

Supplement: Supplementary file 1 — Additional file 1: Figure S1. Ranking of genotypes in the German ‘Idared’ × Malus ×robusta 5 population ordered by degree of infection 20–60 days after inoculation of floral clusters with Ea222_JKI. All data (2011 to 2013 and 2015 to 2017) available for a genotype were averaged for the mean score. Different colours indicate the percentages of the scores 0–6 for the floral clusters of a genotype. [file 12870_2019_2154_MOESM1_ESM.pdf]
