# Supplementary material for: Mapping of fire blight resistance in Malus ×robusta 5 flowers following artificial inoculation
Source: BMC Plant Biol. 2019 Dec 2;19:532. doi: 10.1186/s12870-019-2154-7 (PMC6889339; doi:10.1186/s12870-019-2154-7)
Supplement: Supplementary file 2 — Additional file 2: Figure S2. Significant differences between mean resistance scores of individual years in the German ‘Idared’ × Malus ×robusta 5 population. Only genotypes tested in all years of the respective period were utilized for the analysis. The significance level is α = 0.05. a. Period 2011 to 2013. b. Period 2015 to 2017. c. Period 2011 to 2017 [file 12870_2019_2154_MOESM2_ESM.pdf]

Figure S2: Significant differences between mean resistance scores of individual years. Only genotypes tested in all years of the respective period were regarded. The significance level is α = 0.05

a. Period 2011 to 2013

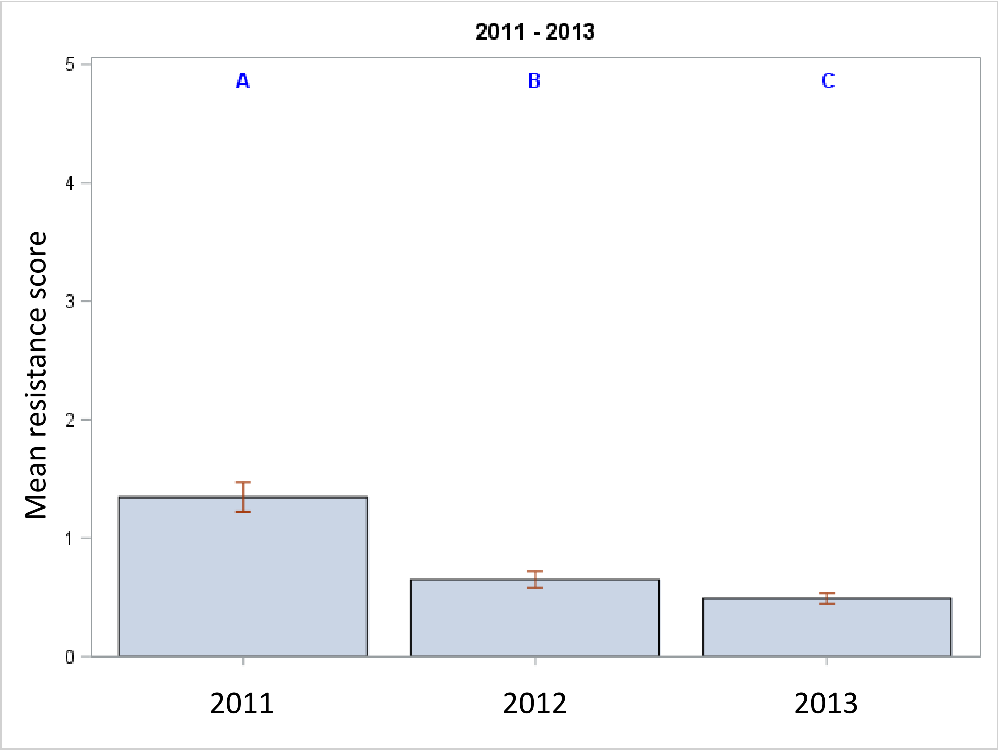

Year	mean	significance		
2011	1.345	A		
2012	0.647		B	
2013	0.489			C

b. Period 2015 to 2017

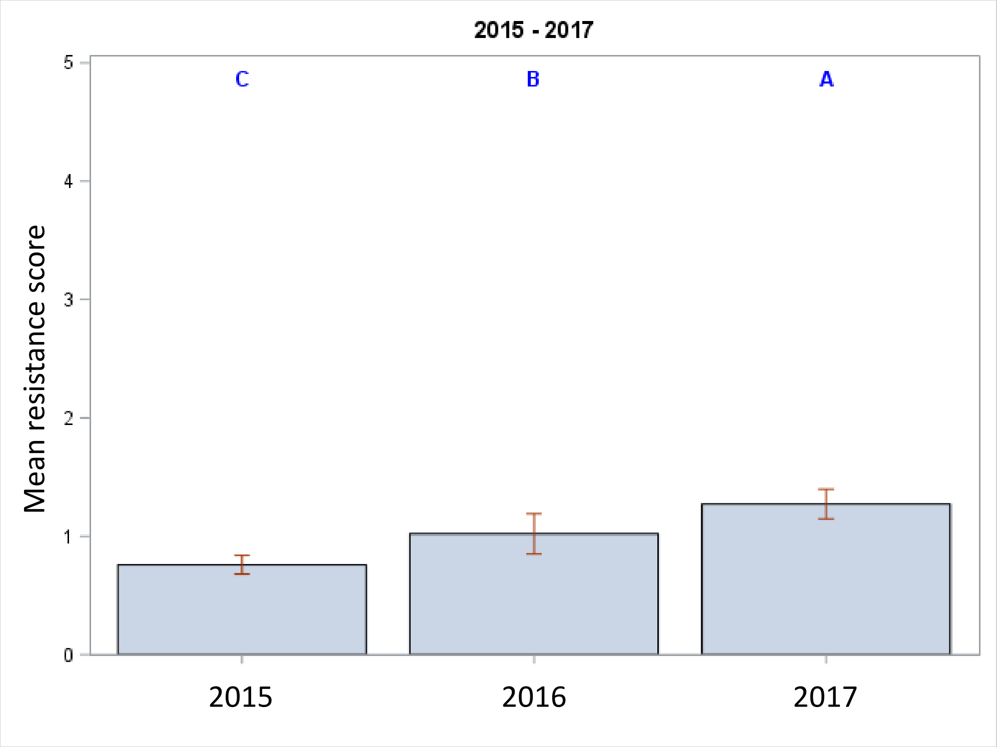

Year	mean	significance		
2015	0.759	A		
2016	1.020		B	
2017	1.271			C

c. Period 2011 to 2017

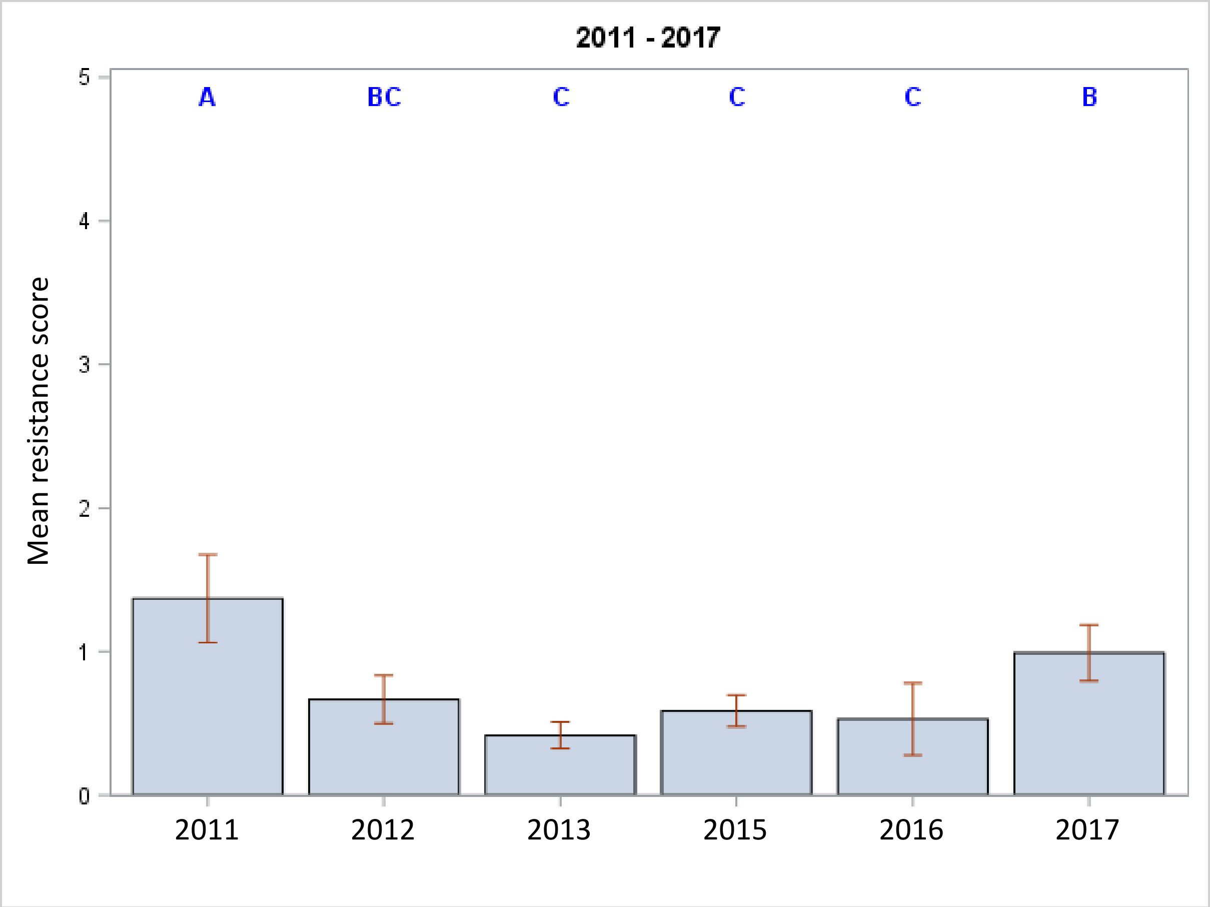

Year	mean	significance		
2011	1.370	A		
2012	0.666		B	C
2013	0.416			C
2015	0.587			C
2016	0.529			C
2017	0.990		B	
