# Supplementary material for: Mapping of fire blight resistance in Malus ×robusta 5 flowers following artificial inoculation
Source: BMC Plant Biol. 2019 Dec 2;19:532. doi: 10.1186/s12870-019-2154-7 (PMC6889339; doi:10.1186/s12870-019-2154-7)

Figure S3: Significant differences between mean resistance scores of genotypes. Only genotypes tested in all years of the respective period were regarded. Genotypes without any symptom in the respective period were removed from analyses, because of the lack of standard deviation. The significance level is α = 0.05

a. Period 2011 to 2013

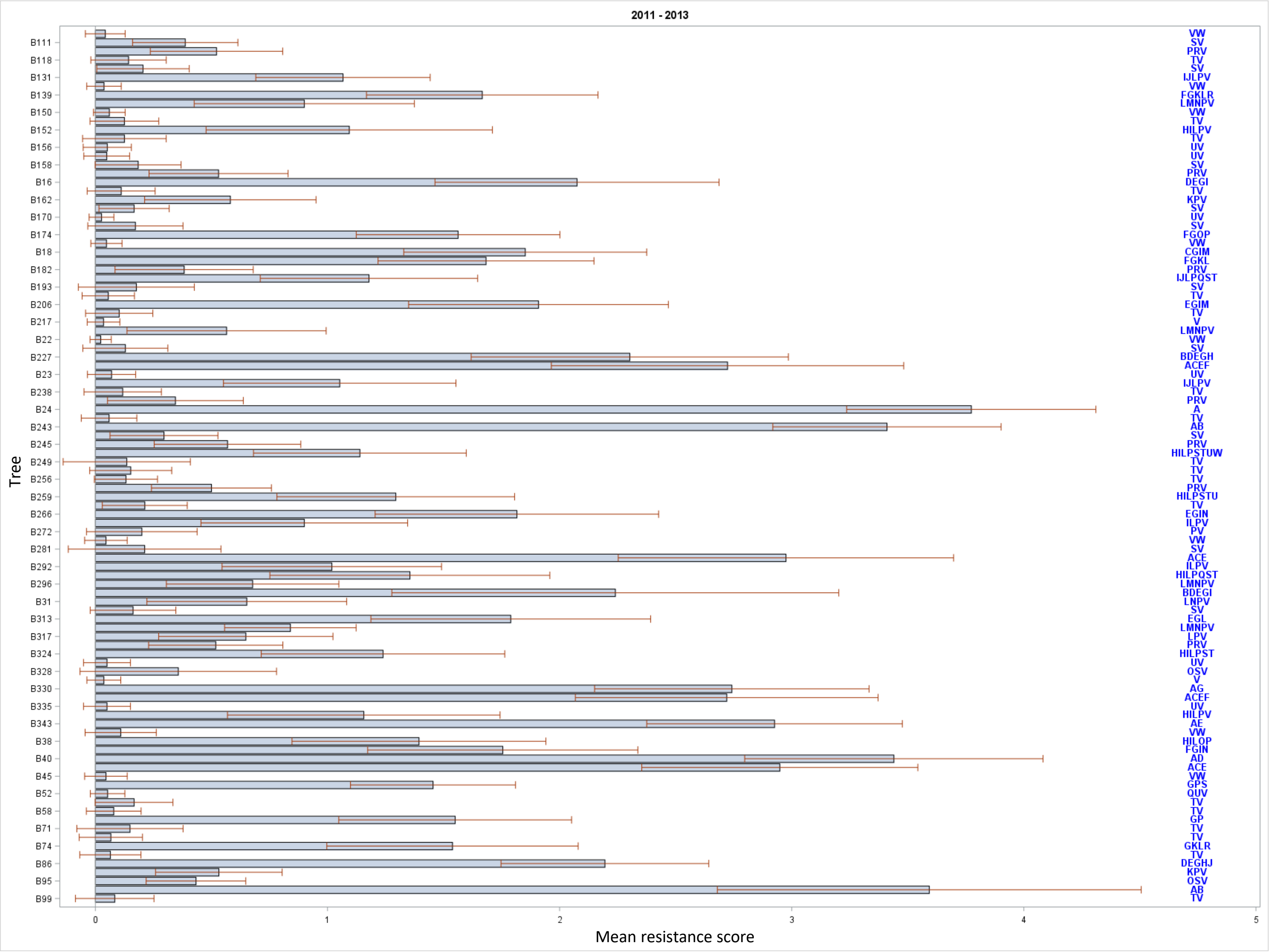

b. Period 2015 to 2017

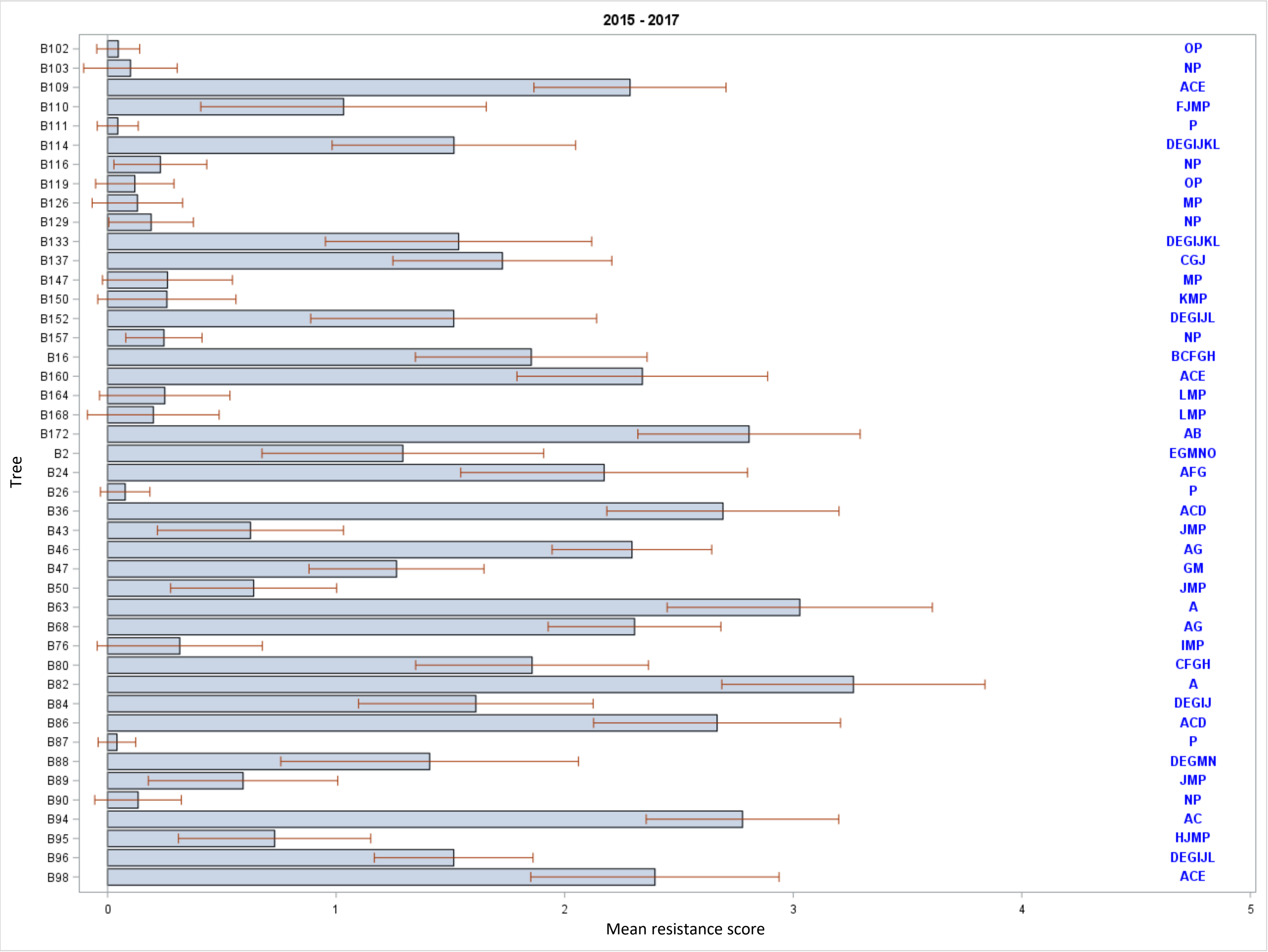

c. Period 2011 to 2017

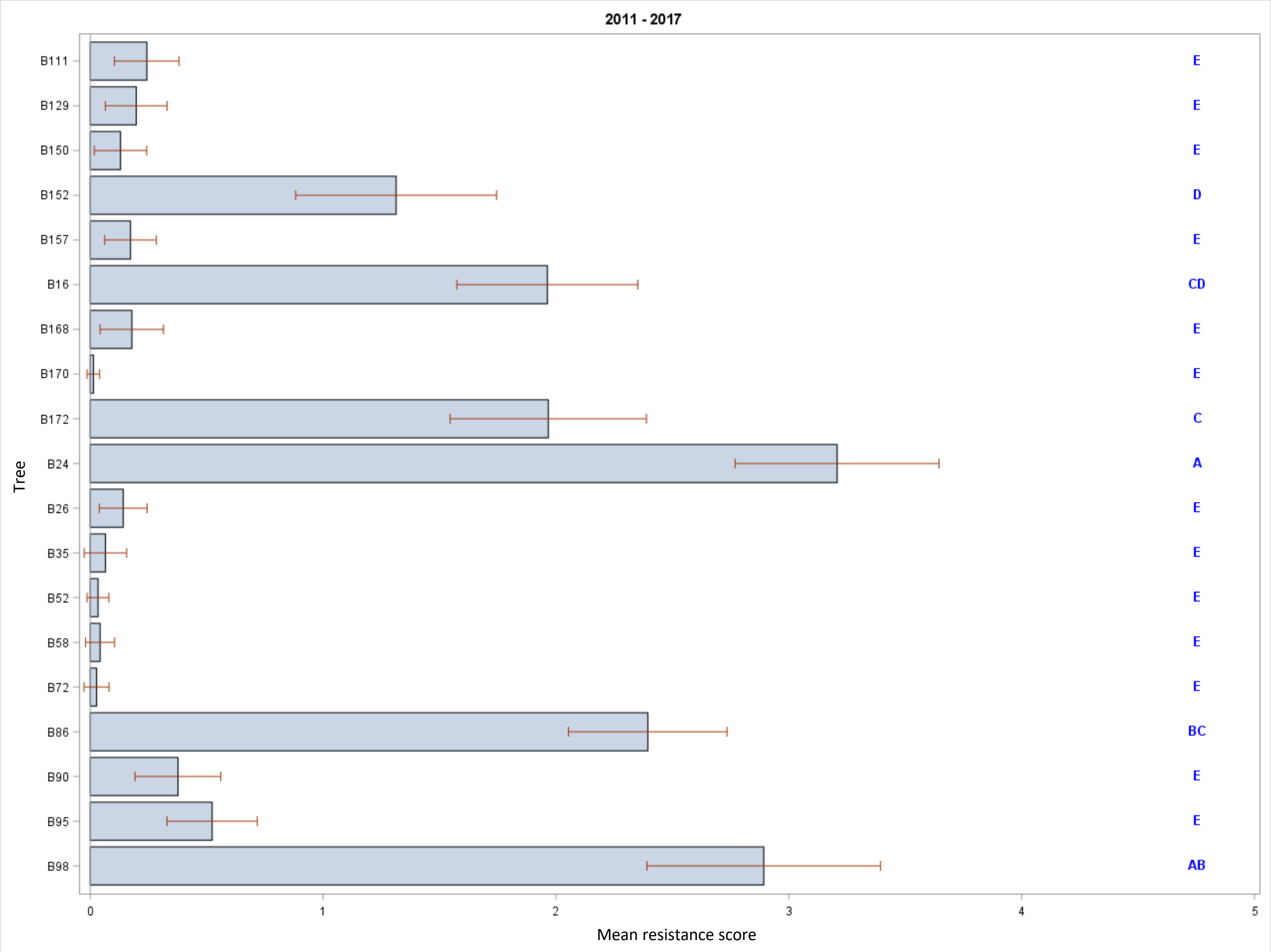

Supplement: Supplementary file 3 — Additional file 3: Figure S3. Significant differences between mean resistance scores of genotypes in the German ‘Idared’ × Malus ×robusta 5 population. Only genotypes tested in all years of the respective period were utilized for the analysis. Genotypes without any symptoms in the respective period were removed from analyses, because of the lack of standard deviation. Different letters on the right side indicate significanet differences at a level of α = 0.05. a. Period 2011 to 2013. b. Period 2015 to 2017. c. Period 2011 to 2017 [file 12870_2019_2154_MOESM3_ESM.pdf]
